# Influence of Sodium Humate on the Growth Performance, Diarrhea Incidence, Blood Parameters, and Fecal Microflora of Pre-Weaned Dairy Calves

**DOI:** 10.3390/ani12010123

**Published:** 2022-01-05

**Authors:** Dong Wang, Zhendong You, Yuanyi Du, Duo Zheng, Haotian Jia, Yun Liu

**Affiliations:** Heilongjiang Key Laboratory of Experimental Animals and Comparative Medicine, College of Veterinary Medicine, Northeast Agricultural University, Harbin 150000, China; wangd931107@163.com (D.W.); you199741@163.com (Z.Y.); duyuanyi0451@163.com (Y.D.); zd18249797629@163.com (D.Z.); jht18804608298@163.com (H.J.)

**Keywords:** dairy calf, sodium humate, diarrhea, growth performance, fecal microbiota

## Abstract

**Simple Summary:**

Diarrhea is a common disease among neonatal calves worldwide and causes serious economic losses in the dairy industry. In most cases, calves receive antibiotics to prevent or treat diarrhea, but the overuse of antibiotics has been linked to the development of antibiotic resistance and negative public health outcomes. Sodium humate (NaH) is a natural product with multiple activities, including antimicrobial, antioxidant, anti-inflammatory, and antidiarrheal properties. This study investigated the effects of different concentrations of NaH on growth performance, diarrhea incidence, serum immunoglobulin and inflammatory cytokines concentration, and fecal microflora in Holstein dairy calves. The results showed that supplementation with 5 g of NaH improved the growth performance, antioxidant and immune status, modulated the fecal microflora, and thereby decreased the diarrhea incidence in dairy calves. This indicated that NaH may be a promising tool for preventing diarrhea in calves.

**Abstract:**

This study aimed to evaluate the effects of the administration of sodium humate (NaH) on the growth performance, diarrhea incidence, and fecal microflora of pre-weaned Holstein calves. In a 53-day experiment, forty healthy newborn female calves were randomly allocated to the following four treatment groups: (1) control (basal diet); (2) 1-gram NaH (basal diet extra orally supplemented with 1 g of NaH dissolved in 100 mL of milk or milk replacer daily); (3) 3-gram NaH (basal diet extra orally supplemented with 3 g of NaH dissolved in 100 mL of milk or milk replacer daily); and (4) 5-gram NaH (basal diet extra orally supplemented with 5 g of NaH dissolved in 100 mL of milk or milk replacer daily). NaH was mixed with milk (d 2–20) or milk replacer (d 21–53). Calves in the 5-gram NaH group had a higher ADG during d 1 to 21 and d 21 to 53 than the other groups did (*p* < 0.05). Fecal scores and diarrheal incidence were significantly lower in the 3-gram and 5-gram NaH groups than the 1-gram NaH and control groups during d 1 to 20 (*p* < 0.05). The serum IgA, IgG and IL-4 concentrations, and T-SOD and T-AOC activities were higher, and the serum IL-6, TNF-α, D-lactic acid, and MDA concentrations were lower in the 5-gram NaH group than the control group (*p* < 0.05). Furthermore, NaH supplementation increased the abundances of *Bifidobacterium* and *Lactobacillus* but decreased the abundance of *Escherichia coli* in feces (*p* < 0.05). These encouraging findings indicated that supplementation with 5 g of NaH effectively improved the immune status, antioxidant capacity, and intestinal beneficial bacteria, and further improved the growth performance and reduced the diarrhea incidence of the pre-weaned dairy calves.

## 1. Introduction

Diarrhea among neonatal calves causes serious economic losses in the dairy industry [1]. Digestive disease and primarily diarrhea are the main cause of calf death [2]. According to one dairy farm survey, 25.3% of calves have diarrhea, with a mortality rate of 4.2% [3]. Antibiotics have long been used in calves as growth promoters and therapeutic agents for diarrhea, but the overuse of antibiotics could cause bacterial resistance and negative public health outcomes. Additionally, the long-term use of antibiotics may disturb the intestinal microflora of calves [4]. The restrictions on antibiotic use further accelerated the seeking for suitable substitutes.

Humic acids (HAs), derived from the decomposition and transformation of decaying organic matter in the soil, are natural organic bioactive agents [5]. As a sodium salt of HAs, sodium humate (NaH) is a multifunctional polymer compound extracted chemically from lignite, weathered coal, and peat, with an average relative molecular mass of 20,000–150,000 and a relative volume mass of 1.33–1.44, containing a variety of alkaloids, vitamins and physiologically active substances [6]. NaH has been traditionally applied for medicinal practice over thousands of years in China. NaH is rich in active groups such as phenolic hydroxyl, carboxyl, sulfhydryl, and carbonyl, and its antimicrobial, antioxidant, anti-inflammatory, and antidiarrheal activities were reported [7]. HAs and NaH have been used orally to treat dyspepsia, diarrhea, and acute intoxication in swine and poultry [8,9]. Murbach et al. (2020) [10] demonstrated the nontoxicity of NaH by in vitro and in vivo tests. In growing-finishing pigs, supplementation with HAs improved growth performance, immune status, and decreased diarrhea incidence [11]. The growth-promoting and antioxidant effects of NaH have also been confirmed in broilers [12]. Moreover, Chudoba-Drozdowska et al. (2000) [13] evaluated the effects of brown coal, HAs, and their mixture on calf health and blood indexes. However, information on the effects of NaH as a dietary supplement for dairy calves is lacking.

The current study sought to investigate the effects of NaH supplementation on the growth performance, diarrhea incidence, and blood parameters related to the immune and antioxidant status and fecal microflora of Holstein dairy calves. We hypothesized that supplementation with NaH can improve growth performance, immune status, and antioxidant capacity and modulate intestinal microflora while reducing diarrhea incidence in pre-weaned dairy calves.

## 2. Materials and Methods

The experimental protocol was approved by the Ethics Committee of Northeast Agricultural University (Harbin, China). The study was conducted at Harbin Modern Farming (Harbin, China). NaH (purity, 75%) was provided by the Institute of Coal Chemistry, Chinese Academy of Sciences (Taiyuan, China). It consisted of 75% humic acid (dry basis), 20.52% burning residue (dry basis), 14.22% water (air dry basis), and 4.48% water soluble substances (dry basis) according to an analysis report of the product. Different extraction processes may affect the yield of NaH and the concentration of humic acid, but the composition and biological activity of NaH are not affected by extraction processes.

### 2.1. Animals, Housing, and Management

Calves were fed 4 L of colostrum by bottle within 2 h of birth and then transferred to individual pens containing straw. Twice daily at 08:30 a.m. and 4:30 p.m., the calves were fed 4 L of pasteurized milk from d 2 to 20, 4 L of milk replacer from d 21 to 36, 3 L of milk replacer from d 37 to 43, 2 L of milk replacer from d 44 to 51, and 1.5 L of milk replacer until weaning (d 53). The milk replacer used in this study contained lactose ≥ 40%, CP ≥ 22%, crude fat ≥ 19%, water ≤ 4.0%, ash ≤ 8.0%, and crude fiber ≤ 0.3%; 150 g of powder was mixed with pasteurized water to make 1 L. A starter was fed to the calves from d 3. All calves had free access to water and starter during the entire experimental period. The ingredients and chemical composition of the starter are shown in Supplementary Table S1.

### 2.2. Experimental Design and Sample Collection

A total of 40 healthy newborn Holstein female calves of initial body weight (BW, 40.1 ± 1.9 kg) were randomly assigned to the following 4 treatments with 10 replicates: (1) control (basal diet), (2) 1-gram NaH (basal diet extra orally supplemented with 1 g of NaH dissolved in 100 mL of milk or milk replacer daily), (3) 3-gram NaH (basal diet extra orally supplemented with 3 g of NaH dissolved in 100 mL of milk or milk replacer daily), and (4) 5-gram NaH (basal diet extra orally supplemented with 5 g of NaH dissolved in 100 mL of milk or milk replacer daily). In the present study, 0, 1, 3, or 5 g of NaH was mixed with 100 mL of milk (d 2 to 20) or milk replacer (d 21 to 53), respectively. The NaH was administered to each calf from a bottle before feeding milk at 08:30 a.m. The trial lasted for 53 d.

### 2.3. Growth Performance, Fecal Score, and Diarrhea Incidence

The experimental period was divided into 1 to 21 and 21 to 53 days of age. During the experimental period, the feed residues were weighed daily, and the BW of the calves was determined weekly to calculate average daily feed intake (ADFI) and average daily gain (ADG). To ensure consistency, the calves were always weighed at the same time.

The fecal score was monitored daily before the morning milk feed according to the method used by Renaud et al. (2018) [14]. Fresh feces were scored by consistency: 0 = firm; 1 = loose or moderate consistency; 2 = very loose or mild diarrhea; and 3 = watery or profuse diarrhea. Diarrhea was defined as fecal scores ≥ 2 occurring for 2 or more consecutive days. According to the fecal score, diarrhea calves were orally administrated with 2 L of electrolytes (Calf Lyte II, Vetoquinol, Shanghai, China) twice a day, and then intramuscularly injected with Ceftiofur at 0.02 mL/kg of body weight (Zoetis, Shanghai, China) for 3 d. The incidence of diarrhea was calculated according to Renaud et al. (2018) [14]. Diarrhea incidence (%) = Number of diarrhea calves × Diarrhea days/ (Number of calves × Test days) × 100. The health conditions and behavior of the calves were recorded daily.

### 2.4. Blood Parameters

Blood samples were obtained from the jugular vein of each calf on d 54 before the morning milk feed. Samples were collected in two 10-milliliter vacuum tubes, one containing ethylene diamine tetra acetic acid (EDTA) anticoagulant and the other without anticoagulant. The samples in the tubes containing anticoagulants were used to determine total red blood cell (RBC) and white blood cell (WBC) counts and hemoglobin (HGB) (Mindray BC-5300, Shenzhen, China). The samples without anticoagulant were centrifuged for 15 min at 3000× *g*, 4 °C. (Eppendorf 5810R, Eppendorf AG, Hamburg, Germany), and the serum supernatants were stored at −80 °C for subsequent analysis. The concentrations of serum total protein (TP), glucose (GLU), and blood urea nitrogen (BUN), and activities of serum alanine aminotransferase (ALT) and alkaline phosphatase (ALP) were analyzed according to the manufacturer’s instructions (Jian cheng Bioengineering Institute, Nanjing, China). Serum IgG, IgM, IgA, insulin-like growth factors (IGFs), growth hormone (GH), inflammatory cytokines (IL-4, IL-10, IL-6, and TNF-α), diamine oxidase (DAO), and D-lactic acid (D-lac) were analyzed using ELISA kits (Jingmei Biotechnology Co, Ltd., Yancheng, China). Serum antioxidant indexes, including total antioxidant capacity (T-AOC), total superoxide dismutase (T-SOD) activity, glutathione peroxidase (GSH-Px) activity, glutathione (GSH) activity, and malondialdehyde (MDA) content were analyzed according to the manufacturer’s instructions (Jian cheng Bioengineering Institute, Nanjing, China).

### 2.5. Fecal Microflora Populations

On day 54, fecal samples were collected from each calf before the morning feeding using sterile gloves. Fecal samples were immediately frozen at −80 °C until further analysis. Bacterial total DNA was extracted from rectum contents using a TIANamp Stool DNA Kit (Tiangen Biotech Co. Ltd., Beijing, China) according to the manufacturer’s instructions. The concentration and purity of the extracted DNA were determined by using a NanoDrop-ND2000 Spectrophotometer (Thermo Fisher Scientific, Inc, Waltham, Massachusetts, USA) and agarose gel electrophoresis. The primers were commercially synthesized by Sangon Biotech (Shanghai, China), and the sequences are presented in Table 1. The abundances of total bacteria, *Bifidobacterium*, *Bacillus*, *Lactobacillus*, and *Escherichia coli* (*E. coli*) were estimated using a real-time quantitative PCR (qPCR) with an SYBR^®^ Premix EX 193 TaqTM II (TaKaRa, Biotechnology Co, Lt., Dalian, China) and a LightCycler 480 system (Roche Molecular Systems Inc., Pleasanton, CA, USA). To quantify the target bacteria, specific standard curves were constructed, and the number of bacterial copies was calculated according to Chen et al. (2013) [15]. The bacteria copies were expressed as log_10_ cells/g of digesta for statistical analysis.

### 2.6. Statistical Analysis

All data were analyzed by one-way ANOVA using the GLM procedure of SAS (SAS Institute, Cary, NC, USA, 2001). All data were analyzed at the level of individual calves, and the normality of the data and homogeneity of variance was checked before statistical analysis. Statistically significant effects were analyzed using Tukey’s HSD test. Differences between treatments were declared significant at *p* < 0.05. Statistical significance was set at *p* < 0.05.

## 3. Results

### 3.1. Growth Performance and Diarrhea Incidence

The effects of NaH supplementation on the growth performance and diarrhea incidence of calves are shown in Table 2. Supplementation with 5 g of NaH increased the ADG of calves during d 1 to d 20 and d 21 to d 53 compared with the control group (*p* < 0.05). However, the BW and ADFI were not affected by NaH supplementation throughout the experiment (*p* > 0.05). The fecal score was lower in the 3-gram and 5-gram NaH groups than the control group during d 1 to d 20 (*p* = 0.02). Furthermore, the diarrhea incidence of calves was 35.7, 29.3, 20.6, and 18.6% at 1 to 20 days of age and 16.3, 14.9, 11.1, and 10.3% at 21 to 53 days of age in the control, 1-gram NaH, 3-gram NaH, and 5-gram NaH groups, respectively.

### 3.2. Blood Parameters

The blood parameters and erythrocyte and leukocyte count of the calves are presented in Table 3. NaH supplementation did not affect the WBC, RBC, HGB, GLU, TP, ALT, ALP, BUN, GH, or IGFs during the experimental period (*p* > 0.05).

### 3.3. Serum Immunoglobulin, Inflammatory Cytokine Levels, and Antioxidant Status

As shown in Table 4, serum IgA (*p* = 0.01) and IgG (*p* = 0.04) were higher in the 5-gram NaH group than the control group. In addition, the higher serum IL-4 and lower serum IL-6, TNF-α, and D-lac contents were observed in the 5-gram NaH group compared with the control group (*p* < 0.05), whereas the serum IgM, IL-10, and DAO concentrations were unchanged (*p* < 0.05). NaH supplementation also affected the serum T-SOD and T-AOC activities and MDA content (*p* < 0.05), whereas the serum GSH and GSH-Px activities were unchanged (*p* > 0.05). Thus, the optimum concentration of NaH for increasing the serum immunoglobulin concentrations and antioxidant enzymes activities was 5 g.

### 3.4. Fecal Microflora

The results of the analysis of the fecal microflora of the calves are presented in Table 5. The abundance of *E. coli* was lowest in the 5-gram NaH, followed by the 3-gram NaH, 1-gram NaH, and control groups. The abundances of *Bifidobacterium* and *Lactobacillus* increased with an increasing NaH concentration. Specifically, the abundance of *Bifidobacterium* was highest in the 5-gram NaH group (*p* = 0.03); the abundance of *Lactobacillus* was higher in the 5-gram NaH group than in the control group (*p* < 0.05). By contrast, the abundances of total bacteria and *Bacillus* were not affected by NaH supplementation.

## 4. Discussion

Diarrhea often occurs in pre-weaned calves and results in growth retardation, low feed efficiency, and high mortality [2]. Calf diarrhea is attributed to both infectious and non-infectious factors. Multiple enteric pathogens are involved in the development of this disease. Although the dairy industry has made great improvements with herd management, animal facilities and care, feeding and nutrition, and the timely use of biopharmaceutics, calf diarrhea is still problematic due to the multi-factorial nature of the disease [1]. NaH is a multifunctional polymer compound extracted from lignite, weathered coal, and peat, which consists of humic acid, burning residue, water, and water-soluble substances [6]. Humic acid is the active component that contributes to the bioactivity of NaH. NaH possesses antidiarrheal, antimicrobial, anti-inflammatory, and growth-promoting properties [7]. We evaluate the effects of administration with NaH on the growth performance and diarrhea incidence of pre-weaned Holstein calves. The results of this study showed that NaH administration mitigated diarrhea and tended to increase the ADG of pre-weaned calves. This is similar to the results of Wang et al. (2008) [21], who observed that including 5 g of NaH improved the growth performance by enhancing nutrient digestion and absorption in finishing pigs, and Ozturk et al. (2012) [22], who found that dietary supplementation with 1 or 1.5 g/kg of HAs improved the ADG, feed intake, and feed conversion ratio in broilers. Importantly, diarrhea is the main factor of growth retardation in calves. The intestinal barrier function is closely associated with diarrhea [23]. The beneficial effects of NaH on the intestinal integrity have been found in broilers, possibly through a decreased intestinal permeability and an increased intestinal viscosity [24]. Trckova et al. (2017) [7] indicated that dietary supplementation with NaH reduced the incidence and duration of diarrhea among weaned piglets. In addition, Kaevska et al. (2016) [9] observed that the fecal scores and diarrhea incidence of weaned piglets were decreased by dietary inclusion with NaH when challenged with enterotoxigenic *Escherichia coli*. Consistently, we found that calves supplemented with 3 g or 5 g of NaH had a lower fecal score and diarrhea incidence from d 1 to d 20. It was reported that the intestinal barrier integrity and mucosal maturation could be reflected by the serum concentrations of DAO and D-lac [25]. The results in the current study revealed that calves supplemented with 3 g or 5 g of NaH had lower serum D-lac concentrations, indicating its beneficial effect on intestinal barrier integrity. To sum up, these observations indicated that the increase in the ADG of the calves supplemented with NaH may be attributed to their improved intestinal barrier function and reduced diarrhea incidence.

On the other hand, the health status of the animals could be reflected by hematological indices and serum metabolite levels. These parameters were within the normal ranges in the current study [26,27]. The results of present study showed that NaH supplementation had no effects on the hematological indices and serum metabolites of pre-weaned calves. The immunomodulatory property of NaH was observed in rats and weaned piglets [28,29]. The concentration of serum immunoglobulin is an important indicator for immune function, which could protect the body against infections and inhibit inflammatory reactions [30]. A previous study found that dietary NaH supplementation increased the antibody titers and immunoglobulin concentration in broilers [31]. This observation provides evidence that its inclusion with 5 g of NaH notably increased the serum concentrations of IgA and IgG in the pre-weaned calves. Interestingly, some investigators revealed that the amount of intestinal beneficial bacteria is associated with the immune function of the host [32]. For instance, Xu et al. (2021) [33] found that the increased concentrations of serum IgG and IgA were positively correlated with the increased abundance of intestinal *Lactobacillus*. Furthermore, the by-products produced from *bifidobacterial* carbohydrate metabolism act as vectors that directly and indirectly trigger the mucosa-associated immune cells to enhance the immune system of the host [34,35]. In the present study, we found that calves supplemented with NaH had a higher concentration of immunoglobulin in the serum and abundances of *Bifidobacterium* and *Lactobacillus* in the fecal samples. Further research on the modulatory effects of NaH on the intestinal microbiota and immune response is required.

Furthermore, inflammatory cytokines, such as TNF-α, IL-6, and IL-1β, play key roles in inflammatory processes [36]. HAs may inhibit the production of pro-inflammatory cytokines. Van Rensburg (2009) [37] indicated that the anti-inflammatory effect of HAs may be exerted by inhibiting classical pathway activation, pro-inflammatory cytokine (TNF-α, IL-1β, and IL-6) release, and promoting lymphocyte proliferation. The results of this study found that the administration of 5 g of NaH reduced the serum IL-6 and TNF-α concentrations in pre-weaned calves, indicating the underlying anti-inflammatory activity of NaH. In addition, the body’s antioxidant defense levels are another important indicator for health status [38]. Changes in the diet or environment of animals can result in oxidative stress [39]. In the current study, NaH supplementation significantly improved the serum T-SOD and T-AOC activities and decreased the serum MDA concentration in the calves, suggesting that NaH may enhance the antioxidant status of calves by increasing the antioxidant enzyme activities. Although data on the antioxidant activity of NaH are limited, the effects of HAs have been widely reported. For instance, Mao (2019) [40] observed that dietary supplementation with 0.6 or 1 g/kg of HAs enhanced the T-SOD and GSH-Px activities and decreased the MDA concentration in the serum of juvenile broilers. Vašková et al. (2011) [41] suggested that HAs contribute to antioxidant defense by scavenging free radicals. Based on these observations and our results, we speculate that the anti-inflammatory and antioxidant capacity of NaH found in this study may be attributed to increased antioxidant enzymes activities and reduced pro-inflammatory cytokines release.

Intestinal microflora helps to improve nutrient utilization and maintain intestinal barrier function and immunity status in animals [42]. A stable gastrointestinal microbiome is critical for optimal growth performance and the immune status of calves [43]. Factors such as diet, age, environment, and management can influence the intestinal microbiota composition [44]. Previous studies have widely reported the modulatory effects of NaH on microbial communities. For example, Domínguez-Negrete et al. (2019) [45] indicated that dietary supplementation with NaH increased the abundance of *Lactobacillus* and decreased quantities of harmful parasites in the intestine of broilers. Furthermore, in piglets, Macri et al. (2016) [46] demonstrated that the administration of NaH could prevent diarrhea by reducing the fecal enterotoxigenic *E. coli* population. The results of the present study indicated that supplementation with 3 g or 5 g of NaH significantly increased the intestinal beneficial bacteria *(Bifidobacterium* and *Lactobacillus*) while decreasing the *Escherichia coli* in the feces of pre-weaned calves. It is well known that *Bifidobacterium* and *Lactobacillus* could produce a variety of biologically active substances, such as short-chain fatty acids (SCFAs), lysozyme, bacteriocin, and antibacterial peptides, which improve animal growth performance by enhancing nutrient utilization, inhibiting the colonization of pathogenic bacteria, and maintaining the balance of intestinal microflora [47,48]. These preliminary results indicated that NaH may have beneficial effects on favorably modulating the intestinal microbiota of calves.

## 5. Conclusions

NaH inclusion effectively decreases diarrhea incidence and improves growth performance in pre-weaned Holstein calves by enhancing their antioxidant and immune status and modulating intestinal microflora, and the optimal concentration of NaH was 5 g.

## Figures and Tables

**Table 1:** Specific primer sequences for bacteria.

Item ^1^	Sequence (5′-3′)	Product Size, bp	References
TB	F: CGGCAACGAGCGCAACCC R: CCATTGTAGCACGTGTGTAGCC	146	Denman and McSweeney. (2006) [16]
*Bif*	F: GATTCTGGCTCAGGATGAACGC R: CTGATAGGACGCGACCCCAT	230	Cleusix et al. (2010) [17]
*Bac*	F: GCAACGAGCGCAACCCTTGA R: TCATCCCCACCTTCCTCCGGT	92	Torrallardona et al. (2007) [18]
*Lac*	F: AGCAGTAGGGAATCTTCCA R: CACCGCTACACATGGAG	341	Wang et al. (2014) [19]
*E. coli*	F: CATGCCGCGTGTATGAAGAA R: CGGGTAACGTCAATGAGCAAA	96	Sapountzis et al. (2020) [20]

Abbreviations: F, forward; R, reverse. ^1^ TB = Total bacteria; *Bif* = *Bifidobacterium*; *Bac* = *Bacillus*; *Lac* = *Lactobacillus*; *E. coli* = *Escherichia coli*.

**Table 2:** Effects of supplementation with different NaH concentrations on the growth performance and diarrhea incidence of pre-weaned Holstein calves.

Item ^1^	NaH Concentration				SEM ^2^	*p*-Value
	0	1 g	3 g	5 g		
BW, kg						
d 0	41.7	39.8	39.6	39.4	0.675	0.628
d 21	52.4	51.8	53.3	55.1	1.050	0.740
d 53	78.7	79.2	80.1	85.2	1.456	0.390
d 0 to 21						
ADFI, g	104.8	108.6	127.1	142.9	0.007	0.296
ADG, g	509.5 ^b^	571.4 ^ab^	652.4 ^ab^	747.6 ^a^	0.035	0.045
d 21 to 53						
ADFI, g	1191.4	1197.9	1215.7	1274.3	0.019	0.438
ADG, g	821.9 ^b^	856.3 ^ab^	837.5 ^ab^	940.6 ^a^	0.022	0.047
Fecal score						
d 1 to 20	2.3 ^a^	1.8 ^ab^	1.6 ^b^	1.5 ^c^	0.071	0.022
d 21 to 53	1.6	1.6	1.5	1.4	0.06	0.746
Diarrhea incidence, %						
d 1 to 20	35.7	29.3	20.6	18.6		
d 21 to 53	16.3	14.9	11.1	10.3		

^a–c^ Means within a row with different letters differ significantly (*p* < 0.05). ^1^ BW = body weight; ADFI = average daily feed intake; ADG = average daily gain. ^2^ SEM = standard error of the mean.

**Table 3:** Effects of supplementation with different concentrations of NaH on the blood parameters including erythrocyte and leukocyte counts of pre-weaned Holstein calves.

Item ^1^	NaH Concentration				SEM ^2^	*p*-Value
	0	1 g	3 g	5 g		
WBC, 10^9^/L	9.92	10.92	9.66	9.88	0.760	0.949
RBC, 10^12^/L	9.44	9.21	9.89	9.76	0.143	0.340
HGB, g/L	100.2	98.8	102	106.2	1.667	0.456
TP, g/L	56.75	57.31	59.08	56.71	0.534	0.395
ALT, U/L	12.88	11.67	14.82	14.86	1.534	0.892
ALP, U/L	342.75	375.75	393.19	382.03	16.523	0.787
GLU, mmol/L	6.8	6.91	7.63	7.65	0.193	0.112
BUN, mmol/L	7.95	6.69	6.8	8.47	0.344	0.184
GH, ng/mL	5.23	5.17	5.2	5.36	0.060	0.759
IGFs, U/L	11.16	10.88	11.23	11.8	0.224	0.593

^1^ WBC = white blood cells; RBC = red blood cells; HGB = hemoglobin; TP = total protein; ALT = alanine aminotransferase; ALP = alkaline phosphatase; GLU = glucose; BUN = blood urea nitrogen; GH = growth hormone; IGFs = insulin-like growth factors. ^2^ SEM = standard error of the mean. Enzyme activity unit = the amount of enzyme that is able to turn over 1 μmol of substrate in 1 min under standard conditions.

**Table 4:** Effects of supplementation with different concentrations of NaH on serum immunoglobulin and inflammatory cytokine concentrations and antioxidant status of pre-weaned Holstein calves.

Item ^1^	NaH Concentration				SEM ^2^	*p*-Value
	0	1 g	3 g	5 g		
IgA, µg/mL	69.13 ^b^	71.64 ^ab^	79.41 ^ab^	80.54 ^a^	2.010	0.014
IgG, µg/mL	985.02 ^c^	997.25 ^bc^	1114.96 ^b^	1128.50 ^a^	25.267	0.046
IgM, µg/mL	69.13	70.39	70.73	70.67	0.380	0.460
IL-4, ng/L	49.13 ^b^	52.47 ^ab^	61.55 ^a^	62.25 ^a^	2.155	0.038
IL-6, ng/L	8.20 ^a^	7.43 ^a^	7.77 ^a^	6.38 ^b^	0.265	0.042
TNF-α, ng/L	203.85 ^a^	185.85 ^ab^	188.56 ^ab^	164.68 ^b^	4.536	0.030
IL-10, ng/L	19.43	20.07	20.59	21.20	0.332	0.299
DAO, pg/mL	196.05	188.87	177.64	168.41	5.126	0.249
D-lac, µg/L	219.27 ^a^	206.77 ^ab^	193.89 ^b^	189.83 ^b^	4.165	0.018
GSH, µmol/L	64.70	67.72	65.90	72.77	1.627	0.344
GSH-Px, U/mL	63.34	62.86	67.25	67.92	1.638	0.737
T-SOD, U/mL	43.70 ^b^	43.94 ^b^	47.08 ^ab^	55.67 ^a^	2.016	0.049
T-AOC, U/mL	5.97 ^b^	6.11 ^b^	6.18 ^ab^	6.35 ^a^	0.050	0.023
MDA, nmol/mL	2.33 ^a^	2.26 ^ab^	2.21 ^ab^	2.10 ^b^	0.036	0.041

^a–c^ Means within a row with different letters differ significantly (*p* < 0.05). ^1^ IgA = immunoglobulin A; IgG = immunoglobulin G; IgM = immunoglobulin M; IL-4 = interleukin 4; IL-10 = interleukin 10; IL-6 = interleukin 6; TNF-α = tumor necrosis factor α; DAO = diamine oxidase; D-lac = D-lactic acid; GSH = glutathione; GSH-Px = glutathione peroxidase; T-SOD = total superoxide dismutase; T-AOC = total antioxidant capacity; MDA = malondialdehyde. ^2^ SEM = standard error of the mean. Enzyme activity unit = the amount of enzyme that is able to turn over 1 μmol of substrate in 1 min under standard conditions.

**Table 5:** Effects of supplementation with different concentrations of NaH on the fecal microbiota of pre-weaned Holstein calves.

Item ^1^ log_10_ Cells/g Digesta	NaH Concentration				SEM ^2^	*p*-Value
	0	1 g	3 g	5 g		
TB	11.91	11.8	11.89	11.9	0.071	0.945
*Bif*	6.53 ^b^	6.98 ^ab^	7.16 ^ab^	7.63 ^a^	0.141	0.035
*Bac*	7.37	7.3	7.59	7.67	0.130	0.751
*Lac*	6.55 ^b^	6.71 ^ab^	7.22 ^ab^	7.58 ^a^	0.176	0.036
*E. coli*	7.62 ^a^	7.05 ^ab^	6.53 ^b^	6.46 ^b^	0.135	0.004

^a,b^ Means within a row with different letters differ significantly (*p* < 0.05). ^1^ TB = total bacteria; *Bif* = *Bifidobacterium*; *Bac* = *Bacillus*; *Lac* = *Lactobacillus*; *E. coli* = *Escherichia coli*. ^2^ SEM = standard error of the mean.

## Data Availability

Not applicable.

## Supplementary Materials

The following supporting information can be downloaded at: https://www.mdpi.com/article/10.3390/ani12010123/s1, Table S1: Ingredients and chemical composition of the starter concentrate diet.
