# Supplementary material for: Influence of Sodium Humate on the Growth Performance, Diarrhea Incidence, Blood Parameters, and Fecal Microflora of Pre-Weaned Dairy Calves
Source: Animals (Basel). 2022 Jan 5;12(1):123. doi: 10.3390/ani12010123 (PMC8749607; doi:10.3390/ani12010123)
Supplement: Supplementary file 1 [file animals-12-00123-s001.zip › animals-1439170-supplementary.pdf]

Supplementary Table S1. Ingredients and chemical composition of the starter concentrate diet

**Ingredient**	**Content (%)**	**Chemical composition**[2]	**Content (%)**
Corn	40.74	DM	87.78
Soybean meal	35.00	EE	4.35
Wheat bran	2.8	CP	23.56
Cottonseed meal	6.8	ADF	5.71
Molasses	4.0	NDF	9.82
Wheat meal	7.8	Ash	3.73
$CaCO_3$	1.63	Ca	0.81
Soybean oil	0.80	Phosphorus	0.49
NaCl	0.10		
$CaHPO_3$	0.10		
MgO	0.07		
Selenium yeast	0.02		
Premix[1]	0.14		
Total	100.00		

[1]The premix provided the following per kg of diet: Fe 206.74 mg, Cu 3.49 mg, Zn 108.81 mg, I 0.60 mg, Se 0.44 mg, Mn 79.99 mg, Co 0.36 mg, VA 9000 U, VD 24000 U, VE 47.22 mg.

[2] Analyzed values.
